# “Meeting people where they are”: Could mobile health clinics be a solution to primary care access challenges faced by newcomers to Canada?

**DOI:** 10.1016/j.dialog.2026.100366

**Published:** 2026-10-19

**Authors:** Sujane Kandasamy, Yusra Naqvi, Kashmira Parikh, Russell J. de Souza, Matthew Y.W. Kwan, Brian McKenna, Diana Sherifali, Lathusha Sritharan, Gita Wahi

**Affiliations:** a Mary Heersink School of Global Health and Social Medicine, Department of Medicine, Faculty of Health Sciences, McMaster University, Ontario, Canada; b Department of Child & Youth Studies, Brock University, St. Catherine's, Ontario, Canada; c Department of Health Research Methods, Evidence & Impact, Faculty of Health Sciences, McMaster University, Ontario, Canada; d Population Health Research Institute, McMaster University, Hamilton, Ontario, Canada; e Department of Family Medicine, McMaster University, Hamilton, Ontario, Canada; f School of Nursing, McMaster University, Hamilton, Ontario, Canada; g Department of Pediatrics, McMaster University, Hamilton, Ontario, Canada

**Keywords:** Mobile health clinic, Newcomers, Primary care access, Type 2 diabetes, Health equity, Community-based screening, RE-AIM framework

## Abstract

**Purpose:**

Mobile health clinics (MHCs) are an emerging approach to improving healthcare access for underserved populations by delivering care directly within communities. This study reports preliminary findings from the "Reach" component of the SCORE! Family Health Van, a community-based intervention designed to evaluate the feasibility of providing culturally responsive type 2 diabetes (T2D) education and risk screening to newcomers in Hamilton, Ontario.

**Methods:**

A cross-sectional descriptive study was conducted using data collected during 31 Family Health Van outreach visits. Participants were recruited through convenience sampling at community-based pop-up events. Community ambassadors provided culturally responsive diabetes education, age-appropriate T2D risk screening, and co-designed educational toolkits. Guided by the RE-AIM framework, this preliminary analysis focused on the "Reach" and early Implementation domains using descriptive statistics.

**Results:**

31 outreach visits resulted in engagement across 34 community location encounters and the recruitment of 72 participants (68 adults, 4 youth). All participants completed diabetes risk screening. Among adults, 45 (66.2%) were classified as low risk, 15 (22.1%) as moderate risk, and 8 (11.8%) as high risk for T2D. High-risk participants (*n* = 8) were provided information regarding follow-up care. Beyond formal recruitment, over 170 additional community members engaged in informal diabetes education and health promotion activities.

**Conclusion:**

Preliminary findings demonstrate the feasibility of using a community-based mobile health approach to engage newcomer populations in culturally responsive diabetes education and screening. These early findings support continued co-development and evaluation of the SCORE! Family Health Van and may inform future mobile health clinic models aimed at improving equitable access to preventive care.

## Highlights

• A community-based mobile approach that reaches diverse newcomer populations.
• 72 participants completed diabetes risk screening across 31 outreach visits.
• Eight adults at high diabetes risk were identified and provided information about follow-up care.
• The model demonstrated feasibility for culturally responsive diabetes screening.
• Findings support future evaluation using the RE-AIM framework.

## 1 Purpose

Mobile health clinics (MHCs) embody the principle of meeting people where they are, providing timely, community-based healthcare in informal and accessible settings, such as open community spaces (including parking lots), parks, grocery stores, residential complexes, and shopping centers [1]. These clinics minimize structural and systemic barriers related to access and transportation by delivering services in familiar environments and fostering trust among underserved populations. Their flexibility to pivot allows them to respond to both chronic and emergent needs across diverse contexts.

For centuries, MHCs have operated globally in geographically isolated areas, refugee camps, and regions affected by war or natural disasters [2], [3]. During the war in Ukraine, MHCs managed chronic disease care for older adults while in Jordanian refugee camps, maternal and newborn health became the focus [4]. Following floods in Myanmar, MHCs integrated disease prevention education into emergency relief [4]. These examples highlight MHCs as essential stopgaps in areas where traditional healthcare infrastructure is limited or absent. The collaborative nature of MHCs brings together healthcare professionals, NGOs, governments, corporate sponsors, and community leaders [5], [6], enhancing cultural responsiveness, trust, and access. This is especially critical for communities that have historically experienced social and structural discrimination [7].

In Canada, the role of MHCs has expanded to reach populations underserved by the current healthcare system. These flexible clinic models are increasingly being used to provide culturally relevant primary care, chronic disease screening, and health promotion services. A 2022 national review identified 29 primary care MHCs across Canada, including 11 in Ontario [8]. This number continues to grow, with recent models like Toronto's Parkdale Queen West Mobile Health Clinic serving neighborhoods with limited access to specialized care facilities and providers [7]. Current MHCs in Canada cater to unhoused communities, Indigenous communities, racialized youth, and immigrants. They offer a range of services including sexual and mental health care, harm reduction, and chronic disease management. Approximately recent environmental scan by our research group found that of the approximately 161 mobile health units operating across Ontario, only two specifically serviced newcomers and refugees [9].

MHCs align with various global and local health priorities and help address Sustainable Development Goal (SDG) 3 (Good Health and Well-being) through improving access to essential health services for underserved populations, and SDG 10 (Reduced Inequalities) by addressing health disparities and increasing access to preventive care and early screening among marginalized groups [10]. Because collaboration is an integral component of a successful MHC to bring together healthcare providers, local community members, and community partners, SDG 17 (Partnerships for the Goals) is also reflected in their approach, with SDG 11 (Sustainable Cities and Communities) embedded within procedural outputs of meeting people where they are and embedding this approach into existing community spaces [10].

Despite their growing presence and value, very few MHCs have been intentionally designed to meet the specific needs of newcomers to Canada. Newcomers often face intersecting barriers to care: lack of knowledge about the system, language barriers, transportation, cultural differences, and mistrust stemming from previous trauma and experiences [11]. For those without a primary care provider, these obstacles can delay both preventive and chronic care, worsening existing health inequities. For example, a study conducted by Adeji et al. (2020) further highlights the importance of addressing type 2 diabetes (T2D) among immigrant populations [12]. Using data from the 2014 Canadian Community Health Survey, this study found that long-term visible minority immigrants had approximately 2.3 times the odds of developing T2D compared with Canadian-born white individuals and approximately 2.1 times the odds compared with long-term white immigrants. These findings suggest that the burden of T2D varies by both racial/ethnic background and duration of residence in Canada, highlighting the importance of accessible, culturally responsive approaches to diabetes prevention, screening, and health education among newcomer communities [12].

Globally, the number of international migrants reached 304 million in 2024, almost double the total in 1990 [13]. As a major destination for resettlement, Canada must be ready to support an increasingly diverse population with healthcare models that are flexible, accessible, and equity-driven. MHCs, especially those built with cultural and linguistic responsiveness, are well-positioned to meet this need. Compared to virtual models of care, MHCs provide face-to-face care, helping address language and system navigation challenges, while reducing transportation and time-related barriers experienced by newcomers [7]. They are cost-effective, can potentially reduce emergency care burden, and foster relationships by integrating services in trusted environments for the communities they serve [1]. By pairing medical services with culturally and lingustically concordant educational tools, they support individuals in taking an active role in their health.

To address these gaps in partnership with community members and partners, the SCORE! Family Health Van project was developed. Phase 1 of the SCORE! Family Health Van research program was designed to determine the feasibility of delivering culturally responsive T2D education and risk screening to newcomer communities through a mobile health clinic approach. The broader intervention was guided by the RE-AIM framework [14]. This paper reports a preliminary analysis of the "Reach" component of the intervention using early program data, examining the extent to which the program engaged diverse participants across community settings, and the risk profile of those reached. Future analyses will evaluate the remaining RE-AIM outcomes, including Effectiveness, Adoption, Implementation, and Maintenance, to assess longer-term impact and sustainability.

## 2 Methods

### 2.1 Study design and setting

This cross-sectional descriptive study reports preliminary findings from the “Reach” component of the SCORE! Family Health Van RE-AIM evaluation. This project is a community-based mobile health clinic conceptualized and developed as an ancillary project, within the SCORE! (Strengthening Community Roots: Anchoring Newcomers in Wellness and Sustainability) program [15]. This broader intervention was co-designed to promote newcomer health and wellness, with the Family Health Van being developed in response to priorities identified through engagement with the local Hamilton community. Specifically, the prioritization of T2D emerged through community discussions and engagement facilitated with the SCORE! Community Advisory Action Board (CAAB), comprised of newcomer families and community partner organizations, and provided strategic guidance for the broader SCORE! research program. This community-engaged approach informed the identification of T2D as a priority for community health promotion and the development of an intervention focused on high-quality diabetes education, risk screening, and prevention among newcomer communities. Once identified, we worked more closely with interested community members and partners to seek feedback on the initial approach, to co-design the pilot study, and develop the approach to subsequent co-design workshops.

Phase 1 of the Family Health Van study seeks to evaluate the appetite for the provision of evidence-based diabetes education, screening, and health promotion directly to communities through pop-up events in natural entry points such as parks, community centers, and newcomer hubs. This research study received ethics approval from the Hamilton Integrated Ethics Board (Project ID: 17729).

Staffed by trained community ambassadors representing the linguistic diversity of the communities served (e.g., Arabic, Punjabi, Urdu, Spanish, French, English, etc.), we provide aesthetically-polished knowledge mobilization toolkits that are practical and culturally relevant, and designed to improve diabetes awareness, health literacy, and access to preventative care.

### 2.2 Data collection

Participants were recruited through convenience sampling during Family Health Van outreach visits conducted independently and in collaboration with local community organizations and events. Adults (≥18 years) provided written informed consent prior to participation, while participants under 18 years of age engaged in capacity-based consent or assent and/or parental consent.

Community ambassadors provided diabetes education, facilitated completion of diabetes risk screening, and distributed the educational toolkits. Data collected included participant age group, sex/gender categories recorded as part of the screening tool, preferred language, diabetes risk screening outcomes, and referral recommendations. We also collected participant feedback on the educational materials. In addition to formal study recruitment, informal community engagement activities were documented to capture the broader reach of program outreach.

### 2.3 Preliminary analysis

Outcomes were examined using the RE-AIM framework (Reach, Effectiveness, Adoption, Implementation, and Maintenance) [14]. As this paper presents preliminary findings from the pilot phase, analyses focused on the "Reach" and early Implementation domains of the framework. Outcomes included the number and types of community settings engaged, participant recruitment, diabetes screening completion, identification of individuals requiring follow-up care, and referral pathways. Descriptive analyses were conducted to summarize community outreach activities, participant characteristics, screening outcomes, and referral patterns.

## 3 Results

### 3.1 Community reach

Preliminary findings for this analysis were drawn from 31 site visits conducted across diverse community settings throughout Hamilton, Ontario. These outreach visits resulted in engagement across 34 community location encounters, as some outreach sessions began and concluded at different locations due to operational considerations (e.g., weather-related relocation).

### 3.2 Participant characteristics

A total of 72 participants were formally recruited across the 31 outreach visits, representing an average of 2.3 participants per visit and a median of 1 (IQR: 0–4). Participants included 68 adults (94.4%) and four youth (5.6%). Sex/gender categories collected through the CANRisk screening tool indicated that 64.7% were female and 35.3% were male.

Participants represented diverse newcomer and racialized communities, including individuals identifying as South Asian, Black/Afro-Caribbean, and East Asian backgrounds. Program delivery occurred in multiple languages, with English, Urdu, and Arabic being the most frequently requested.

Beyond formal study participation, over 170 community members engaged in informal diabetes education and health promotion discussions, demonstrating broader community reach beyond recruited participants.

### 3.3 Diabetes screening and referral outcomes

All 72 participants completed diabetes risk screening. Among the 68 adults screened, 45 (66.2%) were classified as low risk, 15 (22.1%) as moderate risk, and 8 (11.8%) as high risk for T2D based on the CANRISK assessment [16]. Participants classified as high risk (*n* = 8, 11.8%) were provided information about follow-up care.

Participant recruitment varied across community settings. Structured environments, including libraries, schools, and organized community events, generally yielded higher recruitment than outdoor settings such as parks, where weather and participant availability occasionally limited engagement (Fig. 1, Fig. 2).

**Fig. 1:**
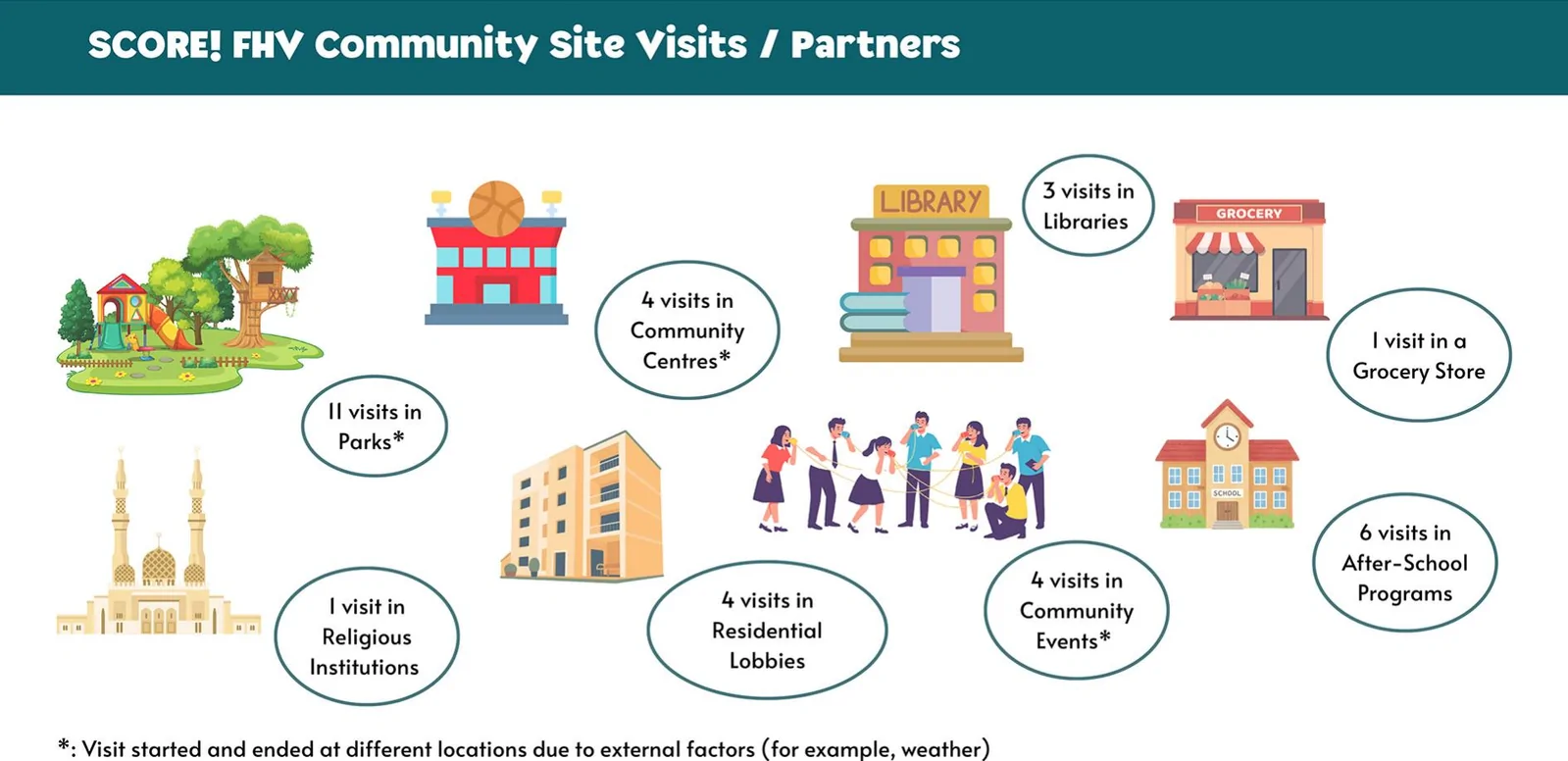
Distribution of community location encounters (*n* = 34) engaged through the SCORE! Family Health Van across Hamilton, Ontario: Community settings included parks (*n* = 11), after-school programs (*n* = 6), community centers (*n* = 4), residential apartment lobbies (*n* = 4), community events (*n* = 4), libraries (*n* = 3), religious institutions (*n* = 1), and grocery stores (*n* = 1). Thirty-one outreach visits resulted in 34 community location encounters because one outreach session was relocated due to weather conditions, and several visits involved more than one community location.

**Fig. 2:**
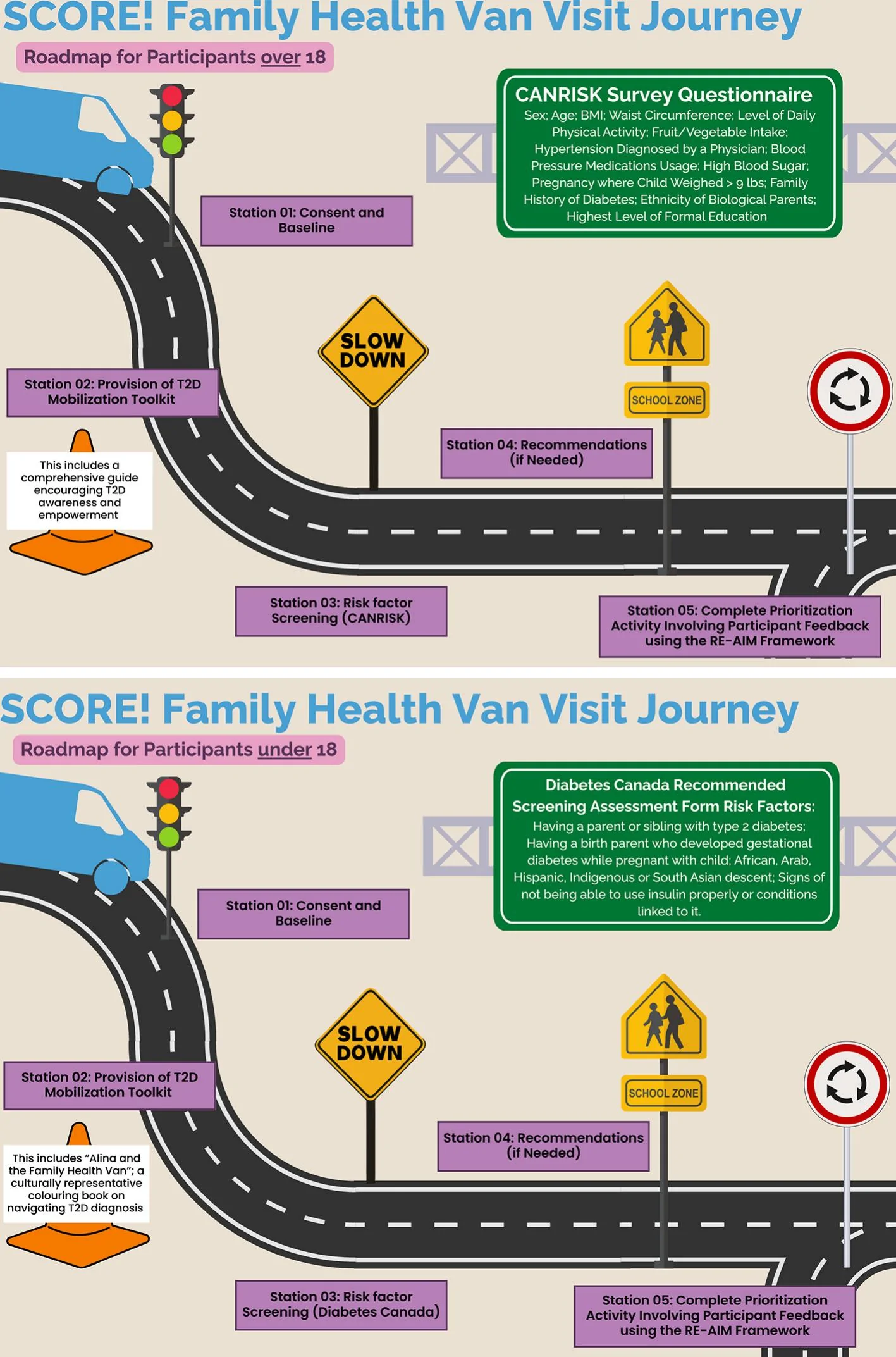
SCORE! Family Health Van participant journey: Overview of the participant pathway for individuals under and over 18 years of age. The visit comprised five stages: (1) informed consent (or assent with parental consent) and baseline data collection; (2) distribution of a culturally relevant Type 2 diabetes (T2D) educational toolkit; (3) diabetes risk screening using age-appropriate tools (Diabetes Canada recommendations for youth and the CANRISK questionnaire for adults); (4) tailored health recommendations and referrals, where appropriate; and (5) collection of participant feedback to inform ongoing program refinement using the RE-AIM framework.

## 4 Conclusion

As the project progresses, we will synthesize the findings from Phase 1, which will guide Phase 2 activities. Phase 2 of this project will seek to co-design community-led health promotion initiatives related to T2D screening, education, and prevention. These will be community-directed and may include diverse components that can be applied using a mobile approach (e.g., walking groups, culturally-reflective physical activities, cooking workshops, and healthcare navigation support). The aim also includes the co-development of “living toolkits” that evolve alongside community needs. The Family Health will also serve as a platform for systemic change by identifying gaps in traditional healthcare models and piloting how mobile, co-designed approaches can help minimize barriers to access and amplify levers to supports. The long-term goal is to not just meet immediate health needs, but to transform how care is delivered, making it more inclusive, proactive, and human-centered.

## Funding disclosure

This project is funded by a CIHR REDI Award that was awarded to Dr. Sujane Kandsamy.

## CRediT authorship contribution statement

**Sujane Kandasamy:** Writing – review & editing, Writing – original draft, Supervision, Methodology, Funding acquisition, Data curation, Conceptualization. **Yusra Naqvi:** Writing – review & editing, Visualization, Data curation. **Kashmira Parikh:** Writing – original draft, Visualization, Data curation. **Russell J. de Souza:** Writing – review & editing, Methodology, Conceptualization. **Matthew Y.W. Kwan:** Writing – review & editing, Methodology, Conceptualization. **Brian McKenna:** Writing – review & editing, Methodology, Conceptualization. **Diana Sherifali:** Writing – review & editing, Methodology, Conceptualization. **Lathusha Sritharan:** Writing – review & editing, Data curation. **Gita Wahi:** Writing – review & editing, Methodology, Conceptualization.

## Declaration of competing interest

The authors declare that they have no known competing financial interests or personal relationships that could have appeared to influence the work reported in this paper.
