# Supplementary material for: “Meeting people where they are”: Could mobile health clinics be a solution to primary care access challenges faced by newcomers to Canada?
Source: Dialogues Health. 2026 Oct 19;9:100366. doi: 10.1016/j.dialog.2026.100366 (PMC13737730; doi:10.1016/j.dialog.2026.100366)
Supplement: Supplementary file 1 [file mmc1.pdf]

Are you at risk for diabetes?

The following questions will help you to find out if you are at higher risk of having type 2 diabetes. You can have undiagnosed type 2 diabetes without having any obvious warning signs or symptoms. Knowing your risk can help you make healthy choices now that will reduce your risk or even prevent you from developing diabetes.

Please answer the questions as honestly and completely as you can. If you wish, a friend or family member can help you to complete this form. Answer all questions. Enter your scores for each question in the box on the right-hand side and then add them up to calculate your total risk score.

This questionnaire is intended for adults aged *18 to 74 years.

SCORE

1. Select your age group:

O *18-44 years — 0 points
O 45-54 years — 7 points
O 55-64 years — 13 points
O 65-74 years — 15 points

* The original CANRISK tool was specifically created for adult populations aged 40–74. Scores for ages 39 and younger are based solely on the CTFPHC Recommendations on screening for Type 2 diabetes in adults.

AS YOU GET OLDER, YOUR RISK OF DEVELOPING DIABETES GOES UP.

2. Are you male or female?

O Male — 6 points
O Female — 0 points

3. How tall are you and how much do you weigh? Use **Appendix 1 (BMI Chart)** on the last page to answer the question below. Select your BMI group from the following choices:

O White (BMI less than 25) — 0 points
O Light grey (BMI 25 to 29) — 4 points
O Dark grey (BMI 30 to 34) — 9 points
O Black (BMI 35 and over) — 14 points

4. Using a tape measure, place it around your waist at the level of your belly button. Measure after breathing out (do not hold your breath). Then check the box that contains your measurement (note: this is not the same as the "waist size" on your pants).

MEN	WOMEN	
O Less than 94 cm or 37 inches	O Less than 80 cm or 31.5 inches	0 points
O Between 94-102 cm or 37-40 inches	O Between 80-88 cm or 31.5-35 inches	4 points
O Over 102 cm or 40 inches	O Over 88 cm or 35 inches	6 points

BODY SHAPE AND SIZE CAN AFFECT YOUR RISK OF DIABETES.

5. Do you usually do some physical activity such as brisk walking for at least 30 minutes each day? This activity can be done while at work or at home.

O Yes — 0 points
O No — 1 point

This tool is an adapted version of the Canadian Diabetes Risk Questionnaire (CANRISK). This tool was developed by the Canadian Task Force on Preventive Health Care (CTFPHC) in partnership with the Public Health Agency of Canada.

CTFPHC

Public Health Agency of Canada | Agence de la santé publique du Canada

info@canadiantaskforce.ca | www.canadiantaskforce.ca

Copyright (2013), *University of Alberta*

SCORE

6. How often do you eat vegetables or fruits?

O Every day	0 points
O Not every day	2 points

YOUR LEVEL OF PHYSICAL ACTIVITY AND WHAT YOU EAT CAN AFFECT YOUR RISK OF DEVELOPING DIABETES.

7. Have you ever been told by a doctor or nurse that you have high blood pressure OR have you ever taken high blood pressure pills?

O Yes	4 points
O No or don't know	0 points

8. Have you ever been found to have a high blood sugar either from a blood test, during an illness, or during pregnancy?

O Yes	14 points
O No or don't know	0 points

9. Have you ever given birth to a large baby weighing 9 pounds (4.1 kg) or more?

O Yes	1 point
O No, don't know, or not applicable	0 points

HIGH BLOOD PRESSURE, HIGH BLOOD SUGAR, AND PREGNANCY-RELATED FACTORS ARE ASSOCIATED WITH DIABETES.

10. Have any of your blood relatives ever been diagnosed with diabetes? Check ALL that apply. Add your score. Your combined score cannot be more than 8 points (2 points for each category, do not count multiple children or siblings twice).

O Mother	2 points
O Father	2 points
O Brothers/Sisters	2 points
O Children	2 points
O Other	0 points
O No/don't know	0 points

11. Please check off which of the following ethnic groups your biological (blood) parents belong to. Choose only one score, the highest. Do not add mother plus father scores together (your score cannot be more than 11 points for this section).

MOTHER	FATHER		
O	O	White (Caucasian)	0 points
O	O	Aboriginal	3 points
O	O	Black (Afro-Caribbean)	5 points
O	O	East Asian (Chinese, Vietnamese, Filipino, Korean, etc.)	10 points
O	O	South Asian (East Indian, Pakistani, Sri Lankan, etc.)	11 points
O	O	Other non-white (Latin American, Arab, West Asian)	3 points

SOME TYPES OF DIABETES RUN IN FAMILIES.

12. What is the highest level of education that you have completed?

O Some high school or less	5 points
O High school diploma	1 point
O Some college or university	0 points
O University or college degree	0 points

OTHER FACTORS ARE ALSO RELATED TO DEVELOPING DIABETES.

Copyright (2013), *University of Alberta*

Add up your points from questions 1 to 12 to determine your Total Risk Score:

SCORE

Points	Chance	Recommendation
**0-32 POINTS**<br>**LOW TO MODERATE RISK**	1-17% chance of being diagnosed with diabetes over the next 10 years*	The CTFPHC recommends <u>not routinely screening</u> for type 2 diabetes.
**33-42 POINTS**<br>**HIGH RISK**	33% chance of being diagnosed with diabetes over the next 10 years*	The CTFPHC recommends <u>screening every 3-5 years</u> with A1C.†
**43+ POINTS**<br>**VERY HIGH RISK**	50% chance of being diagnosed with diabetes over the next 10 years*	The CTFPHC recommends <u>annual screening</u> with A1C.†

* *The 10-year predictive value of CANRISK has been based on the predictive value of the FINDRISC calculator.*

Recommendations are for screening adults without symptoms of type 2 diabetes using blood tests. These recommendations do not apply to adults already diagnosed with type 2 diabetes, those at risk for type 1 diabetes, or those with symptoms of diabetes.

Symptoms of diabetes include: unusual thirst, frequent urination, weight change (gain or loss), extreme fatigue or lack of energy, blurred vision, frequent and recurring infections, cuts and bruises that are slow to heal, and/or tingling or numbness in the hands or feet.

† WHAT IS A1C?
The A1C test is a simple lab test that reflects your average blood glucose level over the last 3 months. A small blood sample to check your A1C can be taken at any time of the day.

Be informed!

TALK TO YOUR HEALTH CARE PROVIDER ABOUT WHEN AND HOW OFTEN YOU SHOULD BE SCREENED FOR TYPE 2 DIABETES.

This tool is an adapted version of the Canadian Diabetes Risk Questionnaire (CANRISK). This tool was developed by the Canadian Task Force on Preventive Health Care (CTFPHC) in partnership with the Public Health Agency of Canada.

CTFPHC

Public Health Agency of Canada　Agence de la santé publique du Canada

info@canadiantaskforce.ca | www.canadiantaskforce.ca

Copyright (2013), *University of Alberta*
